# Reflexivity in Heideggerian Hermeneutic Phenomenology: The Hermeneutic Phenomenological Circle

**DOI:** 10.1111/jan.16950

**Published:** 2025-04-07

**Authors:** Ken Hok Man Ho, Daphne Sze Ki Cheung, Sharon Bourke, David Edvardsson

**Affiliations:** ^1^ School of Nursing and Midwifery La Trobe University Bundoora Victoria Australia; ^2^ Nethersole School of Nursing The Chinese University of Hong Kong Hong Kong SAR China; ^3^ School of Nursing and Midwifery Deakin University Burwood Victoria Australia; ^4^ Centre for Quality and Patient Safety Research/Alfred Health Partnership, Institute for Health Transformation Deakin University Melbourne Victoria Australia; ^5^ School of Nursing The Hong Kong Polytechnic University Hong Kong SAR China; ^6^ Department of Nursing Swinburne University of Technology Melbourne Victoria Australia

**Keywords:** hermeneutic, loneliness, older adults, phenomenology, qualitative, reflexivity

## Abstract

**Aim:**

To discuss reflexivity for conducting hermeneutic phenomenology and to present the hermeneutic phenomenological circle.

**Design:**

Discussion paper.

**Methods:**

We employed data on the lived experience of loneliness of older adults in residential care homes during the COVID‐19 pandemic to demonstrate the reflexivity required for hermeneutic phenomenology. We showcased a reflective process grounded in Heidegger's notions of Being and temporality to uncover the context of meaningfulness and its temporal influence on being.

**Results:**

We proposed a hermeneutic phenomenological circle encompassing four relations passing between Being, the interpreter and being. The circle was successfully applied to demonstrate the reflexivity required to conduct hermeneutic phenomenology.

**Conclusions:**

Engaging in reflexivity requires the researcher to develop knowledge about the philosophy of hermeneutic phenomenology. The hermeneutic phenomenological circle facilitates researchers in anchoring their reflections in Being and temporality.

**Implications:**

The hermeneutic phenomenological circle serves as a useful tool for researchers/health professionals to learn hermeneutic phenomenology and to facilitate researchers/health professionals at all career stages to engage in reflexivity.

**Summary**

- What is already known?
  ○ Reflexivity is a poorly addressed topic in qualitative research.
  ○ There is no explicit and descriptive account of reflexivity in hermeneutic phenomenology.
- What this paper adds?
  ○ Proposes a hermeneutic phenomenological circle for reflexivity in hermeneutic phenomenology.
  ○ Demonstrates that Being and temporality are anchors for reflexivity in hermeneutic phenomenology.
  ○ Shows that reflexivity is dependent on the paradigmatic and methodological stances of researchers.

## 1 Introduction

‘Any discipline that aims at a deeper understanding of human experience will find that the hermeneutic‐phenomenological method is an exceptional and rigorous research avenue’ (Ayala 2008, 414). Hermeneutic phenomenology is, therefore, popular in nursing research and has strong implications for person‐centred care (Ho and Chiang 2024). Phenomenological approaches are also becoming increasingly popular in medical studies (Larsen et al. 2022; Olmos‐Vega et al. 2023). Hermeneutic phenomenology demands that the researcher engage in deep reflexivity to uncover other possible ways of being (Ho et al. 2017). Braun and Clarke (2019); Braun et al. (2022); Braun and Clarke (2023) suggested that such an analysis is an example of reflexive thematic analysis, which requires a very high degree of reflexivity and theoretical sensitivity to construct a meaning‐unified‐interpretative story, instead of producing a topic summary. In fact, there are guidelines (Olmos‐Vega et al. 2023) or a scoping review (Landy et al. 2016) that describe methods of applying reflexivity in qualitative research. However, there is no standard technical method of using reflexivity in hermeneutic phenomenology, apart from engaging in abstemious reflection as a way to approach hermeneutic phenomenology (Errasti‐Ibarrondo et al. 2019; van Manen 2014). Descriptive accounts of reflexivity in phenomenology are also scare (McNarry et al. 2019). Clinicians and health professional researchers or students may have trouble engaging in reflexivity informed by the philosophical tenets of hermeneutic phenomenology (Ho et al. 2017).

Since hermeneutic phenomenology is primarily based on Heidegger's philosophy of Being and temporality in his masterpiece *Being and Time* (1927/2008), this paper aims to provide an example of the reflexivity surrounding Being and temporality using the published narrative of loneliness among older adults living in residential care homes during the COVID‐19 pandemic (Ho, Mak, et al. 2022). We first explain the concept of Being and being, as well as temporality, and then give an example of reflexivity when phenomenologically analysing loneliness among older adults. We then propose a hermeneutic phenomenological circle and discuss its application in facilitating reflexivity as the underpinning to conducting hermeneutic phenomenology (Ho et al. 2017).

## 2 Background

### 2.1 Being and being

Questioning the Being of being is the most important task for Heidegger in Being and Time (1927/2008). The distinction between the two is fundamental to Heidegger, who emphasises that Being cannot be understood as being (1927/2008). In order to distinguish clearly between Being and being, we have to understand how Heidegger describes our various ways of existing in and interpreting the world. In ‘Heidegger and the Hermeneutic Turn’ (Hoy 2006), ordinary interpretation, that is, being, is distinguished from philosophical interpretation, that is, Being. Ordinary interpretations include the everyday phenomena of entities (e.g., skills, objects, identities), whilst philosophical interpretation, Being, is a reflective approach to working on phenomena to capture a primary understanding of the world (Hoy 2006). Ordinary interpretations more or less automatically change when our projects and needs change. For example, we interpret ourselves as a patient, parent, student or employee according to changes in the situation. These changing identities, as interpretated by an individual according to the demands of the situation, are examples of being (Ratcliffe 2002).

Meanwhile, Being has an ontological significance in that Being is presupposed in all beings (Heidegger 1927/2008). All changing ordinary interpretations (i.e., beings) presuppose the existence of a ‘primary understanding (i.e., Being)’ of the world (Hoy 2006). A primary understanding is not the assertion that facts about the world are uncovered or discovered (1927/2008). Rather, a primary understanding is the grasping of the entire mode of entities. Heidegger (1927/2008) holds that we grasp entities in their webs of relations with other entities. For example, we do not merely see a touch screen and infer that it is a smartphone. Instead, we see a touch screen, together with other functional parts (e.g., Wi‐Fi access, camera), and secondarily abstract a web of relations between these functional parts to meaningfully synthesise the existence of a smartphone. However, it is important to note that if we do not have a primary understanding of the relations between functional parts, we are unable to synthesise a smartphone. As such, the being of the smartphone is already enmeshed in the primary understanding of how functional parts relate to each other. Our understanding of how entities shall relate to other entities, as the context of meaningfulness, form our primary understanding of the world, presupposing that every intelligible being is in a web of relationships. This primary understanding is referred to as pre‐understanding, presumption or fore‐structure in phenomenology (Johnston et al. 2016).

According to Ratcliffe (2002), Being can be understood as constitutive teleology, which is about the presupposed conditions accounting for our purposive commitment to interpret, experience and make sense of the world and ourselves. These presupposed conditions are the shared intelligible context of meaningfulness, as a familiar world, on the basis of which we understand who we are and who we are going to be. Therefore, what Heidegger refers to as Being in human existence can be the context of meaningfulness within which we are already enmeshed, which forms our primary understanding of the world and the self (Heidegger 1927/2008). This is in contrast to the bracketing (also referred to as ‘epoche’) of descriptive phenomenology, where we have to isolate the context of meaningfulness in a phenomenological analysis (McNarry, Allen‐Collinson, & Evans, McNarry et al. 2019; Thomas and Sohn 2023). It is the context of meaningfulness in human existence as an anchor of reflexivity that underpins the basis of hermeneutic phenomenology.

### 2.2 Temporality

In order to perform a hermeneutic phenomenological analysis, we have to further understand the temporal relationship between Being and being in human existence. The temporality is that Being is always ahead of itself (Heidegger 1927/2008), meaning that the context of meaningfulness from the past is always being projected into the future, and directs our everyday ordinary interpretations, as being (Guignon 2006). This is because our understanding of entities is already enmeshed in context and the context is projected into the future, presupposing the intelligibility of being (Guignon 2006). The temporality is best illustrated by Krell, ‘I pursue various possibilities for my future, bear the weight of my own past, and act or drift in the present’ (Krell 2008, 22). However, the possibilities that lie in front of us are not infinite and we are not without constraints when choosing from a range of possibilities. The context of meaningfulness (e.g., a particular historical culture or set of social norms) predefines the range of possible actions that will make sense in one's own situation (Guignon 2006). Therefore, a human knows how to act in a situation at present as presupposed by a context of meaningfulness from the past and foreseeing a consequence of the enactment in the future. The endurance of a context of meaningfulness from the past through to the present and into the future is the temporality (Heidegger 1927/2008). Whilst Being is an anchor for understanding lived experiences, temporality provides the space for this anchoring. As such, Being and temporality form the two anchors for reflexivity in hermeneutic phenomenology.

## 3 Data Sources

Employing published data collected from older adults living in residential care homes between 1 June 2020 and 6 July 2020 during the COVID‐19 pandemic in Hong Kong, I (the first author) shared the reflexive journey of disclosing the context of meaningfulness in which I and the older adults and I were enmeshed. The findings of the study were published. In the study, I ‘uncovered the purpose of functionality as a shared background in the core of their (older adults') identity. When older adults oriented themselves toward the future, a dominant intention ‘to be functional’ guided older adults to interpret their self‐identity and to sustain the integrity of their selves’ (Ho, Mak, et al. 2022). In this article, I demonstrated the reflexivity required for conducting a hermeneutic phenomenological analysis by intertwining data on older adults and my experience of loneliness during the Severe Acute Respiratory Syndrome epidemic.

## 4 Overview of the Issues

Reflexivity is often poorly addressed in qualitative research (Olmos‐Vega et al. 2023). As in many other qualitative reports, reflexivity in the phenomenological findings has not been fully disclosed in manuscripts due to space limitations (Peddle 2021). In fact, the process of practising reflexivity to uncover the context of meaningfulness behind the loneliness of older adults was lengthy and nonlinear. It was an iterative process of reflecting on my own values and assumptions, considering how they have become interwoven with the narratives of older adults, and with Being and temporality as the anchor of this process of reflexivity. As such, we demonstrated the back‐and‐forth reflections that led us to a deeper understanding of the lived experience of loneliness by anchoring reflexivity to Being and temporality. Therefore, our demonstration of reflexivity filled a gap in the poorly addressed topic of reflexivity in hermeneutic phenomenology. The following section serves as an example that demonstrates the exercise of reflexivity by paying attention to Being and temporality in hermeneutic phenomenology.

## 5 Findings

Since first starting my study of loneliness (Ho, Cheung, et al. 2022), comments were made that the interactionist perspective of loneliness is the most frequently used perspective in theories and best fits the everyday concept of loneliness in a contemporary society (De Jong Gierveld et al. 2018). Accordingly, loneliness refers to the unpleasant experience arising from a subjective deficit in the quantity or quality of social relationships (Peplau and Perlman 1982). Grounded in this definition, I conducted two cross‐sectional studies (Ho, Cheung, et al. 2022; Ho et al. 2023) on the loneliness of older adults, which empirically validated the interactionist perspective. I planned another cross‐sectional study to examine the prevalence of loneliness among older adults in RCHs during the COVID‐19 pandemic, based on the assumption that weakened social connections during the social restrictions imposed during the COVID‐19 pandemic would exacerbate the loneliness of older adults in residential care homes. However, it was not practical to collect data from hundreds of older adults during the pandemic. Therefore, I initiated a phenomenological study to explore the lived experience of loneliness of older adults in RCHs during the COVID‐19 pandemic (Ho, Mak, et al. 2022).

Starting from the first interview, I expected to hear about miserable experiences of being isolated without any contact with relatives or friends. However, I was surprised by the first older adult whom I interviewed:

My home is here [the residential care home]. The nurses are very good. They are concerned about my feeling of being trapped every day. They hug me. They talk with me. They encourage me to reach out within this home, of course, wearing a mask (laughed). I don't feel anxious here. I feel loved. (Ho, Mak, et al. 2022, 285)

At that moment, I could not make sense of that statement because, contrary to my assumption, the older adults did not experience loneliness. Therefore, I thought that the quality of the data was poor and decided to keep the data as a negative case for future analysis.

Grounded on my assumption of weakened social connections, I intentionally kept looking for any cues about interpersonal/social disconnections contributing to loneliness among older adults:

Group activities are cancelled, and my family cannot visit me. It's really disturbing to have this free time. I have nothing to do every day, but my mind is not at peace. Every piece of information about COVID‐19 makes me anxious. Without family and group activities, I keep the anxiety within me. I keep silent and bear it. My impression during the past few months is of loneliness. (Ho, Mak, et al. 2022, 284)

At first sight, the above experience disclosed weakened social connections as ‘group activities are cancelled, and my family cannot visit me’. I kept thinking about whether social disconnection is a context of meaningfulness in the loneliness felt by older adults. In hermeneutic phenomenology, it is important to answer how the self is purposively committed and related to others, within the context of meaningfulness across time (van Manen 2014). As such, it was important to ask for what purposes were the older adults socially connected? And on what grounds did the older adults maintain those purposes through being socially connected? In this sense, being socially disconnected may only be an objective situation, so that older adults could not enact those purposes. As such, being socially disconnected was not a context of meaningfulness. Furthermore, the participant did not only attribute loneliness to being ‘without family and group activities’, but also to ‘*bearing anxiety*’. This led me to question, ‘Why was there anxiety in loneliness?’ ‘What is the nature of this anxiety?’ The anxiety may shed light on the purposive commitment of older adults with others in their lived experiences of loneliness when they were socially disconnected.

According to Heidegger (1954/2008), humans tend to lose insights into everydayness. This means that humans are too busy with the mundane tasks of living to satisfy the needs of others. Reflexivity requires researchers to reflect on their own values, assumptions, expectations, choices and actions during the research process (Braun et al. 2022). This is in accord with Heidegger (1927/2008), who stated that the clearing of Being requires us to reflect on our everydayness. To make sense of loneliness alongside ‘*bearing anxiety*’, I therefore reflected on my experience of loneliness during the severe acute respiratory syndrome (SARS) epidemic in Hong Kong in 2003. A researcher's own experience of a phenomenon could be an important source of data to explicate the researcher's assumptions (Johnston et al. 2016). The context of the disease and the atmosphere of the society was similar to the situation during the COVID‐19 pandemic. My experience with SARS facilitated my reflections on loneliness with anxiety and social disconnection.

I was a fresh nursing graduate with around 3 months of clinical experience when I contracted SARS. I was hospitalized in an acute ward for 3 weeks and spent another month in a rehabilitation hospital. No visits were allowed during the period of hospitalization. I was extremely lonely whilst in the acute ward. Although I was surrounded by familiar colleagues (who were either sick or on duty) at that moment, my loneliness involved having to keep my feelings of anxiety to myself. To everyone, I pretended to be optimistic. However, my weeping at night uncovered that I feared that I might be the next one to die, to vanish. As a registered nurse, I logically understood that there was no effective treatment for SARS. What I was waiting for was either to recover naturally by myself or to suffer until I died. Emotionally, I was upset because I was a burden to everyone at that moment and I was unable to achieve more. Although society praised us (i.e., healthcare professionals, particularly those who contracted the disease whilst performing their duties) as heroes, I was unable to feel a sense of pride. Rather, I was ashamed for not able to contribute anything.

My anxiety during the period of SARS uncovered my sense of finitude. I had run out of options in my life. According to Heidegger (1927/2008), experiencing finitude is one of the fundamental characteristics of human existence. However, my awareness of finitude came too early and too drastically for a young adult. Alongside death, it was my first time to be in a situation in which I could do nothing and just receive help. I was struck heavily with the notion that ‘I was ashamed for not being able to contribute anything’, alongside the thought that I was at that moment going through a journey that would possibly lead to a dead end. The words ‘burden’ and ‘contribute’ showed how I purposively related myself to others across time based on my instrumental value to others. This self‐understanding allowed me to more closely approach the context of meaningfulness in loneliness among older adults:

My daughter cares about me. During the lockdown, she phones me every day. However, I am still lonely. My daughter and I are in two parallel worlds. I was smuggled from Mainland China to Hong Kong when I was very young. I built my family on my own and I raised my daughters with my own labour…. The point is that I am the only one to face my Parkinson's [disease]. They cannot solve my problem. I have five roommates and I am the only one who can still walk and eat. However, I am also the only one who will witness myself becoming one of them…. I hope I will get the coronavirus. I am valueless to society and a burden to my daughters. (Ho, Mak, et al. 2022, 284)

The older adult uncovered his own finitude through the quote ‘I am also the only one who will witness myself becoming one of them…’. At this point, the older adults forecasted a dead end without possibilities. As such, I produced a preliminary theme entitled ‘I am deserted by all possible beings as destined by COVID‐19’. However, this theme could not reflect the context of meaningfulness in the lived experience of loneliness across time. It did not show how older adults were purposively committed to the world and to others across time. According to Heidegger (1927/2008), humans can be with others because humans have a relationship to that world. ‘Becoming one of them’, on the one hand, showed a relationship between the older adult and other older adults who were bed‐bound. On the other hand, ‘becoming one of them’ bore the deeper meaning of how the self of the older adults related to the world. Through ‘becoming one of them’, the older adult perceived himself as burdensome and valueless to others. There was a context of meaningfulness projected onto the older adults, in which they were characterised as burdensome and valueless when the individual himself became one of the bed‐bound older adults.

What was the context of meaningfulness veiled behind the words ‘burdensome’ or ‘valueless’ after realising that the situation was finite? I further reflected on my experience with SARS. In contrast to the loneliness that I felt in the acute ward, my experience in the rehabilitation ward was much more positive. It was not about the quality of the caring. It was about my gradual reestablishment of purpose when I related myself to the world. To make contributions was at the centre of my identity. Being able to contribute, I would not be a burden. Expecting a journey of recovery, my world became more possible because I would once again be functional! ‘*Functionality*’ demonstrated utility, as a context of meaningfulness, in which human nature was measured in terms of effects, in order to justify human existence (Heidegger 1946/2008). That context of meaningfulness is pervasive in a modern society (Heidegger 1954/2008). As a young adult at the time, I was less capable of reflecting on my life. ‘*Functionality*’ formed the dominant context of meaningfulness for me to purposively commit to others, to the world. The endurance of functionality was demonstrated through its temporality, where I had to be functional in all my projections into the future (Heidegger 1927/2008). At the time of the SARS epidemic, I could not project my functionality during the period that I spent in the acute ward. Paradoxically, the context of functionality also saved me from extreme loneliness when my condition improved during my stay in the rehabilitation ward. In fact, I was still a non‐functional patient in both situations. Functionality plunged me into loneliness, whilst also saving me from loneliness.

With insight into the context of functionality and its temporal influence, I was more ready to resonate with the loneliness of older adults during the COVID‐19 pandemic:

I built my family on my own and I raised my daughters with my own labour…. I hope I will get the coronavirus. I am valueless to society and a burden to my daughters. (Ho, Mak, et al. 2022, 284)

The endurance of functionality from the past through to the present and into the future guided the older adults to interpret themselves (to build and to raise). However, the functionality also drove older adults to project themselves as ‘unable to be functional’. In contrast to my luck in having the opportunity to recover, the COVID‐19 pandemic and the irreversible functional decline that some older adults experienced uncovered the disconnection between the older adults and the context of functionality. A disconnected commitment in a shared world based on functionality deprived the older adults of their feeling of self‐significance, uncovering their existential loneliness:

I have several children but have ended up living alone in a nursing home [RCH]. This disease (COVID‐19) further keeps me alone. I don't know the reason for surviving. I don't know what I am for…. (Ho, Mak, et al. 2022, 285)

According to Heidegger (1927/2008), the public context of meaningfulness drives humans to interpret their own being in terms of serving external purposes, instead of developing an understanding of who they are or who they are to be. As such, loneliness was uncovered as an indication of a poverty of self‐understanding, such that the older adults lost their sense of the uniqueness of their existence (Costache 2013). Meanwhile, it is also important to review negative cases to deepen our understanding of the relationship between the context of meaningfulness and oneself. Therefore, I revisited the experiences of the first older adult whom I interviewed:

My home is here [the residential care home]. The nurses are very good. They are concerned about my feeling of being trapped every day. They hug me. They talk with me. They encourage me to reach out within this home, of course, wearing a mask (laughed). I don't feel anxious here. I feel loved. (Ho, Mak, et al. 2022, 285)

The context of functionality was not found in the above case. The older adult was not assessed by others or by themselves based on their utilisation. By reviewing those stories containing a minimal level of loneliness, we further developed the insight that functionality was not the only context of meaningfulness in human existence:

During the COVID‐19 pandemic, I plant things in the backyard of the RCH. When I feel lonely, I look at the plants. They grow day by day. It's fulfilling. (Ho, Mak, et al. 2022, 285)

The older adults were able to interpret themselves based on an alternative purposive commitment of being alongside the growth of plants; it was not necessary to be functional for someone or something. This is in accord with the view of Heidegger (1946/2008), who stated that the clearing of Being requires the development of insights outside of the dominant way of thinking. For older adults dwelling in their loneliness, the context of functionality was dominant in their world. When older adults were unable to project themselves from the past through to the present and on to the future according to the dominant context of functionality, they experienced loneliness. As such, the theme was further revised to better reflect the disconnection between oneself and context of meaningfulness across time in loneliness. This is because ‘A deprived sense of self‐significance in a familiar world contributes to older adult's disconnection with prior commitments’ (Ho, Mak, et al. 2022, 283).

## 6 Discussion

We gave an example of the reflexivity involved in conducting a hermeneutic phenomenological analysis by anchoring the reflexivity to Being and temporality (Heidegger 1927/2008). Uncovering the context of meaningfulness as ‘functionality’ allowed me to understand how this primary understanding was projected onto the beings of older adults, leading them into the abyss of loneliness. Although reflexive accounts are an under‐addressed subject in qualitative research in the health disciplines (Peddle 2021; Olmos‐Vega et al. 2023), reflexivity is highly dependent on the paradigmatic and methodological stance taken by researchers (Braun and Clarke 2019). Although various types of reflexive writing (e.g., journal writing, field notes, structured questionnaires) have been identified as examples of the practice of reflexivity (Landy et al. 2016; Peddle 2021), they are generally applicable to all qualitative inquiries. On the one hand, the absence of a guide to reflexivity for hermeneutic phenomenology can impose barriers to the learning of hermeneutic phenomenology and put hermeneutic phenomenology at risk of being practised by caprice. On the other hand, we agree with van Manen (2014) that there are no prescribed steps or methods for conducting hermeneutic phenomenology. Following prescribed steps/methods to conduct phenomenology does not make a study phenomenological.

**FIGURE 1:**
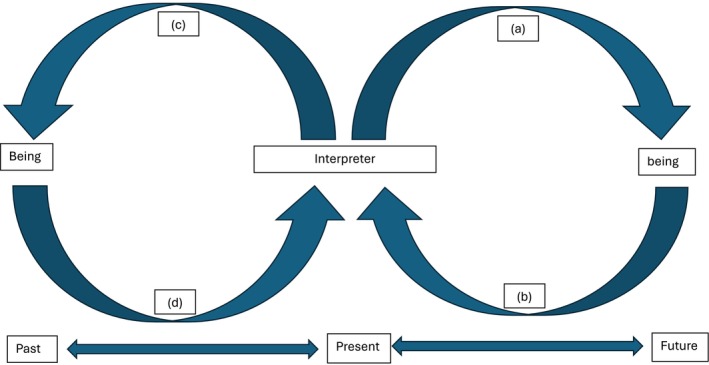
Hermeneutic phenomenological circle.

A starting point for discussion is the hermeneutic circle because it signifies an important methodological process of interpretation and reflection in hermeneutic phenomenology, whilst not being prescriptive. Gallagher (1992, 106) conceptualised the hermeneutic circle as a recurring cycle of four relations passing between tradition, the interpreter and the object: (1) tradition (e.g., history, culture) operates as a fore‐structure of understanding; (2) the interpreter brings the fore‐structure to interpret an object (e.g., a situation, a text, a human); (3) the feedback that resulted from interpreting an object motivates a new projection of meaning (e.g., an alternative interpretation); and (4) the new projection of meaning further challenges and modifies the tradition, thereby changing the fore‐structure of understanding. For Gallagher (1992), there is no escape from the hermeneutic circle because all humans belong to history and to tradition. According to Heidegger (1943/2008), Being operates as a fore‐structure of understanding to direct our interpretation. Therefore, the conceptualisation of the hermeneutic circle (Gallagher 1992) is in accord with the temporality of Being, whereby we enact the context of meaningfulness from the past into the present, and project it into the future (Heidegger 1927/2008). However, the hermeneutic circle (Gallagher 1992) only explicates the process of interpretation by shedding light on the influence of tradition and how traditions are being challenged to reach a new understanding. Within the context of hermeneutic phenomenology, it still does not explicate what is to be reflected in relation to the philosophy of phenomenology. As such, we modified the hermeneutic circle based on Gallagher (1992) to propose the hermeneutic phenomenological circle for the purpose of reflexivity in hermeneutic phenomenology (Figure 1). The hermeneutic phenomenological circle encompasses four relations (a), (b), (c) and (d) passing between Being, the interpreter and being. At the bottom of the circle, temporality is shown as a continuum from the past to the future.

In principle, Being, as the context of meaningfulness, implicitly directs our interpretations of being (Ratcliffe 2002). However, in practice, it is usually the case that we develop an understanding of a being with minimal insight into the context of meaningfulness. This is in accord with Heidegger (1954/2008), who stated that getting lost in being is the first step to approach the clearing of Being. As such, the interpreter (a) purposively relates to and interprets being according to context of meaningfulness. At this moment, the interpreter may or may not have insights into his/her own primary understanding of the phenomenon. However, being is an opening to Being (Heidegger 1927/2008). The being (b) shows itself to the interpreter as ‘it is at first and for the most part—in its average everydayness’ (Heidegger 1927/2008, 59). This implies that the manifestation of being is mostly dominated by a secular understanding of the world. For example, technology is about the means to an end, in which the nature is the application of a method to produce a desired effect (Heidegger 1954/2008). It is one of the dominant contexts of meaningfulness in modern society (Heidegger 1954/2008). It is important for an interpreter to reflect the veiled context of meaningfulness covered by the manifestation of being. As being is a product of interpretation by the interpreter, it is suggested that the interpreter dwell in the language of the participants (Ho et al. 2017) and draw on various source materials (e.g., personal history, documentaries, poets, etc.) for reflection (Johnston et al. 2016) and to uncover the veiled context of meaningfulness. Uncovering the veiled context of meaningfulness will help the interpreter to (c) reflect on the dominance of a context of meaningfulness and motivate the search for alternative contexts of meaningfulness. This opens the interpreter to an awareness of the presence of various possibilities of human existence (Heidegger 1946/2008). As such, the interpreter (d) is able to reflect on the influence on his/her primary understanding and uncover alternate contexts of meaningfulness to operate as alternate fore‐structures in understanding. The whole circle thereby loops again with more diversity in interpretation, showing both the dominant and veiled beings. Meanwhile, the temporality of Being is shown in the back‐and‐forth continuum from the past through to the present and into the future. This continuum reminds the interpreter to reflect on the notion that Being is always ahead of being, whilst being from the future also allows the interpreter to retrospectively reflect on Being.

### 6.1 Application of the Hermeneutic Phenomenological Circle

Using my example of reflexivity in the hermeneutic phenomenological analysis shown above, I am going to explain my process of reflexivity in relation to the hermeneutic phenomenological circle.

From interpreter to being (a): In the beginning, I thought that a manifestation of loneliness, as being, is social disconnection. I also explicitly justified loneliness based on the interactionist perspective, which could be the dominant context of meaningfulness at that moment. The interactionist perspective kept me looking for cues on interpersonal disconnections. At that time, I did not develop any insights into functionality.

From being to interpreter (b): Through dwelling in the language (e.g., anxiety, burden, valueless) of the participant and by continually questioning the nature of the context of the meaningfulness that directs the purposive commitment of older adults with others, the authentic nature of the being of loneliness showed itself to me as not simply consisting of social disconnection. Further reflexivity was facilitated by revisiting the nature of Being and being grounded in Heidegger's philosophy. For example, humans tend to lose insights into their everydayness, and the clearing of Being requires us to reflect on our everydayness (Heidegger 1927/2008; Heidegger 1954/2008). Whilst Heidegger's philosophy provided directions for reflection, my reflexivity on everyday functionality as the context of meaningfulness was facilitated by reflecting on my own experience when placed in a similar context to that of older adults (Johnston et al. 2016). However, it is not unusual for researchers not to have a shared or similar experience with the research participants. We suggest that researchers draw on various materials (e.g., movies, poetry, documentaries, etc.) to expand their horizons. More importantly, this process is an iterative and time‐consuming process, requiring that a great deal of time be devoted specifically to this process.

From interpreter to Being (c): Through uncovering the context of functionality in the purposive commitment of older adults with others and with the world, the disruption of self‐significance grounded in a familiar context of meaningfulness was uncovered as the root problem of the loneliness of older adults in residential care homes during the COVID‐19 pandemic. This opened up an opportunity for me to understand the dominance of functionality in everyday life, and also motivated me to look for alternate contexts of meaningfulness in the lived experiences of older adults during the pandemic.

From Being to interpreter (d): I further looked for cases where older adults did not interpret themselves based on their functions or effects for someone or something. There were cases where older adults were simply being alongside the growth of a plant, and this was already fulfilling. This showed the possibility of having more than one context of meaningfulness. It allowed me to more closely approach the clearing of Being by understanding the ‘possibilities’ of human existence (Heidegger 1946/2008).

The temporality continuum: By understanding the dominance of functionality, researchers can employ the temporality continuum to construct meaning‐unified reflective stories. It is important not to fall into the trap of chronological or objective time when constructing the reflective story. The tenet of the reflective story shall show that Being is always ahead of being, regardless of objective time (Heidegger 1927/2008).

## 7 Strengths and Limitations

The hermeneutic phenomenological circle provides a tangible idea of reflexivity in hermeneutic phenomenology, without departing from Being and temporality. The four relations between Being, interpreter and being are not sequential prescriptive steps. They explicate the relationship between Being, interpreter and being; therefore, researchers can start at any point and jump between any point depending on their understanding of hermeneutic phenomenology. As such, the hermeneutic phenomenological circle is a flexible tool for researchers at any career stage or level of experience in hermeneutic phenomenology. Although we showed the application of the hermeneutic phenomenological circle in a hermeneutic phenomenological analysis, the circle is applicable to all stages of hermeneutic phenomenology.

However, the hermeneutic phenomenological circle only provides one of the many pathways for reflexivity in hermeneutic phenomenology. Using the hermeneutic phenomenological circle still requires the researcher to have some knowledge of Heidegger's philosophy. It is also not an exhaustive explanation of the philosophy of Heideggerian hermeneutic phenomenology. Whilst the hermeneutic phenomenological circle can assist researchers in approaching the clearing of Being, a total clearing of Being can never be achieved because Being encompasses much more than history, tradition, culture and experience (Heidegger 1927/2008). Since reflexivity depends heavily on the paradigmatic and methodological stance of researchers, the hermeneutic phenomenological circle may not be immediately applicable to other qualitative methodologies, including descriptive phenomenology. For example, the concept of bracketing in Husserlian descriptive phenomenology requires researchers to completely isolate their pre‐understanding in a phenomenological analysis, which goes against the philosophy of Heidegger and is impossible in practice (McNarry et al. 2019).

## 8 Conclusion

A hermeneutic phenomenological analysis is a specialised approach, and those conducting it must be very sensitive to the implicit meanings spoken by clients (Ho et al. 2017). Having a deep philosophical understanding of hermeneutic phenomenology is a solid foundation for practising reflexivity in hermeneutic phenomenology. Employing loneliness as an example, we proposed the hermeneutic phenomenological circle to explicate the anchors of reflexivity when conducting hermeneutic phenomenology. The hermeneutic phenomenological circle will serve as a useful tool to ease the barriers to learning hermeneutic phenomenology and to improve the quality of a hermeneutic phenomenological analysis by anchoring the analysis to Being and temporality.

## Author Contributions

K.H.M.H. made substantial contributions to conception and design, or acquisition of data, or analysis and interpretation of data; K.H.M.H., D.S.K.C., S.B. and D.E. were involved in drafting the manuscript or revising it critically for important intellectual content; given final approval of the version to be published. Each author should have participated sufficiently in the work to take public responsibility for appropriate portions of the content; agreed to be accountable for all aspects of the work in ensuring that questions related to the accuracy or integrity of any part of the work are appropriately investigated and resolved. The authors take full responsibility for this article.

## Ethics Statement

Ethics approval is not required.

## Consent

The authors have nothing to report.

## Conflicts of Interest

The authors declare no conflicts of interest.

## Data Availability

The data that support the findings of this study are available from the corresponding author upon reasonable request.
